# Effectiveness of Individual Oral Health Care Training in Hospitalized Inpatients in Geriatric Wards

**DOI:** 10.3390/ijerph20054275

**Published:** 2023-02-28

**Authors:** Stephanie Viebranz, Marco Dederichs, Anja Kwetkat, Ina Manuela Schüler

**Affiliations:** 1 Department of Prosthetic Dentistry and Material Science, Centre for Dental Medicine, Jena University Hospital, 07743 Jena, Germany; 2 Department of Geriatrics and Palliative Care, Klinikum Osnabrück GmbH, 49076 Osnabrück, Germany; 3 Section of Preventive and Pediatric Dentistry, Department of Orthodontics, Centre for Dental Medicine, Jena University Hospital, 07743 Jena, Germany

**Keywords:** dental plaque, oral health care training, denture hygiene, older people, geriatric inpatients

## Abstract

Objective: To investigate the effectiveness of individual oral health care training (IndOHCT) on dental plaque removal and denture cleaning in hospitalized geriatric inpatients. Background: The literature reveals neglect of hygiene and oral care in people aged over 65 years, especially in persons in need of care. Hospitalized geriatric inpatients have poorer dental health than those non-hospitalized. Furthermore, the existing literature reporting on oral healthcare training interventions for hospitalized geriatric inpatients is scarce. Materials and Methods: This pre-post-controlled intervention study dichotomized 90 hospitalized geriatric inpatients into an intervention group (IG) and a control group (CG). Inpatients in the IG received IndOHCT. Oral hygiene was assessed using the Turesky modified Quigley–Hein index (TmQHI) and the denture hygiene index (DHI), at baseline (T0), at a second examination (T1a), and after supervised autonomous tooth brushing and denture cleaning (T1b). The influence of the Mini Mental State Examination (MMSE), Geriatric Depression Scale (GDS), and Barthel Index (BI) scores on oral hygiene was examined. Results: There was no significant plaque reduction on teeth or dentures between T0 and T1a in either group. Between T1a and T1b, plaque reduction on the teeth was more effective in the IG than in the CG (*p* < 0.001). Inpatients with 1–9 remaining teeth removed significantly more dental plaque than inpatients with 10 or more remaining teeth. Inpatients with lower MMSE scores (*p* = 0.021) and higher age (*p* = 0.044) reached higher plaque reduction on dentures. Conclusions: IndOHCT improved oral and denture hygiene in geriatric inpatients by enabling them to clean their teeth and dentures more effectively.

## 1. Introduction

The increased proportion of older people in the general population is expected to increase the need for care in the coming centuries [1]. General diseases occur more frequently in older age, further increasing the need for medical and general care. Hospitalized geriatric inpatients carry a double burden of disease. In addition to age-related disabilities, they suffer from multimorbidity and acute illnesses [2].

Due to the prioritization of acute medical care during hospitalization, little focus is given to oral hygiene and oral health care in the hospital setting [3]. The majority of older people only have complaint-oriented dentist visits [4,5]. Due to the high hospitalization rate of older people, it is possible to reach seniors who otherwise might only visit the dentist for dental pain [6,7].

Oral health has a significant impact on general health [8]. It is associated with the prevalence of systemic diseases, such as renal insufficiency [9], diabetes [10], atherosclerosis [11], and cardiovascular diseases [12]. Furthermore, good oral hygiene can significantly reduce the risk of respiratory infections [13,14]. Poor oral health is also associated with higher frailty [15,16,17] and in-hospital mortality in inpatients [18].

In addition, malnutrition is one of the major problems in the treatment of geriatric inpatients. This is caused by impaired chewing function, which is associated with the consumption of soft and unhealthy food [19]. Oral health and hygiene should be assessed regularly [20] to detect oral problems early. Timely executed preventive and curative treatment reduces the risk of complications and general health deterioration. Individualized and intensive preventive oral care appears to be effective [21,22] in maintaining oral health.

The literature reveals neglect of hygiene and oral care in people aged over 65 years [23], especially in persons in need of care [5]. Since cognitive and fine motor skills decrease with older age [24], it becomes more difficult to perform effective oral hygiene autonomously. Effective oral hygiene is a challenge for inpatients and care providers in geriatric settings [25]. Social and medical changes, lack of dexterity, decreased salivary flow, poor oral hygiene, and wearing dentures are risk factors for increased susceptibility to dental caries [26].

Neglected oral hygiene significantly impairs quality of life. Several studies have described the association of dental caries, periodontal disease, and inadequate dentures with quality-of-life deterioration in older people [27,28,29,30].

Studies focusing on oral hygiene in geriatric inpatients are limited [31,32,33]. Hospitalized geriatric inpatients have poorer dental health than the non-hospitalized [34,35,36]. Hospitalized geriatric inpatients have lower numbers of natural teeth and are more frequently edentulous [31,37].

The aim of the present case-controlled intervention study was to assess the oral health and oral hygiene status of geriatric inpatients and to evaluate the efficacy of dental and denture cleaning training interventions on plaque reduction. Three hypotheses were tested: (1) individualized oral health care training improves the oral hygiene status of geriatric inpatients, (2) age, sex, and oral health status of geriatric inpatients have no influence on plaque removal on teeth and dentures, and (3) the geriatric assessment tests—Mini Mental State Examination (MMSE), Geriatric Depression Scale (GDS), and Barthel Index (BI) have no influence on plaque reduction on teeth and dentures.

## 2. Materials and Methods

This case-controlled intervention study was conducted between 2012 and 2015 at the Department of Geriatric Medicine, Jena University Hospital, Germany. The study protocol was approved by the Ethics Committee of Jena University Hospital, Germany (3277-10/11), and the study was registered with the German Registry of Clinical Trials (DRKS 00004742).

### 2.1. Study Sample

The inclusion criteria for participation in this study were (1) hospitalization at the Clinic of Geriatric Medicine, Jena University Hospital, (2) a mini-mental state examination (MMSE) score ≥ 24 points, and (3) provision of informed consent. Inpatients aged ≥ 80 years are generally classified as geriatric. Inpatients older than 60 years are also hospitalized in geriatric wards if, in addition to multimorbidity, they also suffer from typical geriatric syndromes, such as immobility, fall-related injuries, incontinence, dementia, depression, and malnutrition. Sample size calculation was performed prior to inpatient enrolment. To achieve a statistical power of 80% for proving the superiority of the intervention group (IG) compared with the control group (CG) in plaque reduction, 43 inpatients were required in each group, 32 of whom needed to be dentate.

Of the 2772 geriatric inpatients hospitalized during the study period, 1211 met the inclusion criteria. Of these eligible inpatients, only 165 consented to participate.

**Figure 1:**
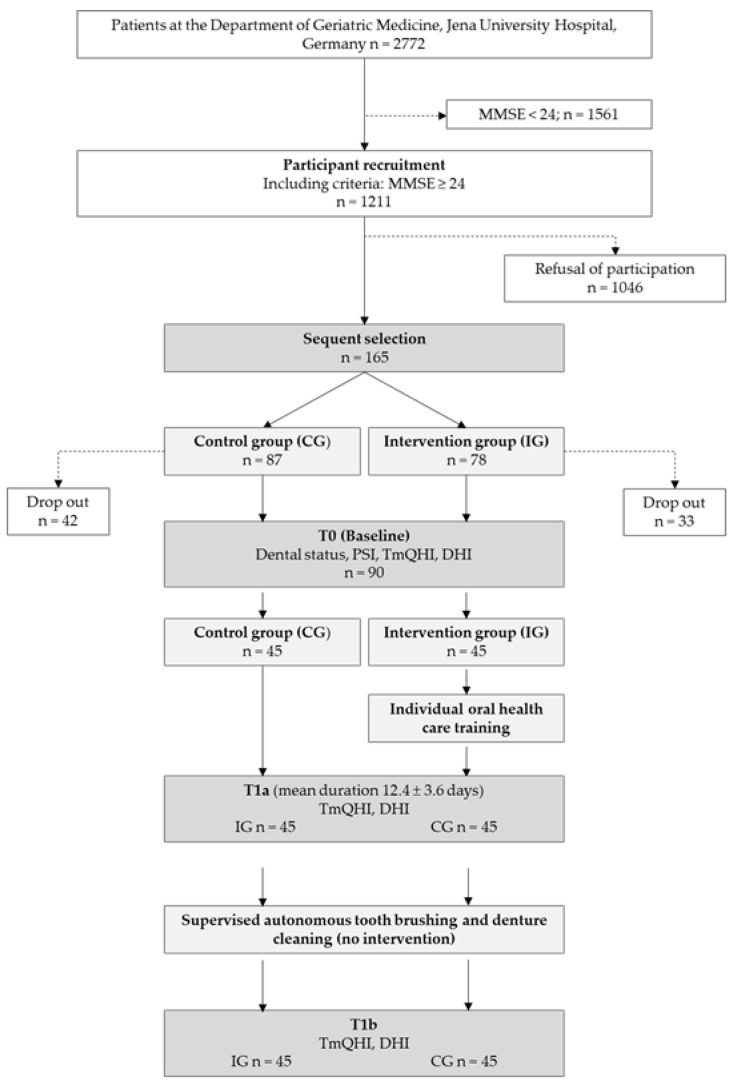
Study design.

Thirty-three and forty-two inpatients dropped out in the IG and CG, respectively, due to death, transfer to other hospitals, withdrawal of consent, quarantine, deteriorated general condition, or lack of willingness to continue participating in the study. The assignment to both groups was sequential. First, inpatients were assigned to the IG until the required number of dentate and edentulous inpatients was reached. Inpatients satisfying the matching criteria for sex and dentition status (dentate/edentulous) were then enrolled in the CG. Finally, data from 45 inpatients, 32 of whom were dentate, were included in each group. All inpatients provided written informed consent to participate and for access to their medical records. The inpatient enrolment procedure is illustrated in Figure 1.

### 2.2. Oral Examinations

Prior to the clinical study, the dental examiners (I.S., B.B., and K.A.) were calibrated by theoretical and practical training. The calibration was performed on six inpatients in a setting comparable to that of the study. Inter-examiner reproducibility was assessed using kappa statistics (k): 0.7 for K.A. vs. B.B. and I.S. vs. K.A. and 0.9 for B.B. vs. I.S.

The first oral examination (baseline; T0) was performed shortly after inpatient admission to the hospital. The examination was conducted by a dentist and an assistant for documentation. The inpatients were examined while sitting or lying on the hospital bed. Complete dental status was assessed using an illuminated mirror (DenLite; Miltex Inc., York, PA, USA) and a standard probe after drying the teeth with cotton rolls. Dental caries was diagnosed according to the WHO standard [38] using the DMFT index. Gingival and periodontal health was evaluated using the periodontal screening index (PSI) [39], using a standard ball-end probe with a light probing force. The highest score for the entire dentition was recorded. Dental plaque was assessed by the Turesky modified Quigley–Hein index (TmQHI) [40] after staining all the teeth with Mira-2-Ton© solution (Miradent Hager and Werken GmbH & Co. KG, Duisburg, Germany). Dentures were only briefly rinsed under running water to remove food residue without brushing or plaque staining. Plaque distribution was evaluated using the denture hygiene index (DHI) [41].

The second (T1a) and third (T1b) assessment was performed in one session shortly before the inpatients’ dismissal from the hospital. The mean time between T0 and T1a was 12.4 ± 3.6 days. At T1a, TmQHI and DHI were recorded. The inpatients were examined for the presence of dental and denture plaque. Subsequently, the inpatient performed oral health care autonomously while the examiner exclusively observed the inpatient. No interventions or assistance were given. Afterwards, at T1b, the plaque parameters TmQHI and DHI were recorded again.

### 2.3. Intervention

After the first oral examination at T0, inpatients in the IG received a one-time individual oral health care training (IndOHCT) from the examiner. This individualized training included oral health information, motivational arguments, and practical exercises of tooth brushing and denture cleaning. The information provided included the inpatient’s oral health and oral hygiene status as well as the etiology and consequences of dental plaque, gingivitis, and caries. The important role of oral hygiene in maintaining good oral and general health, especially in older inpatients with multimorbidity, was explained. Practical training began with staining all teeth with a plaque revealing solution (Miradent Hager and Werken GmbH & Co. KG, Duisburg, Germany) and followed by observing the inpatients’ tooth brushing and denture cleaning as they usually do. Patients were encouraged to use their own tooth and denture cleaning utensils and were not provided with standardized oral hygiene products. The intention was to observe how patients were able to use their usual toothbrushing utensils to identify any motor impairments in their use and to provide guidance and individualized tips on how to use the products more effectively to improve plaque removal. Consequently, oral hygiene products were recommended individually according to the functional and cognitive status of the patient and their use was intensively trained [42]. Insufficiently cleaned tooth surfaces and segments of the dentures were detected collaboratively. For these surfaces and segments, alternative brushing techniques were explored, tested, and training given. Functional limitations of the inpatients, such as reduced manual dexterity, shoulder mobility, and visual acuity, were taken into consideration. If necessary, silicone handles were attached to toothbrushes to compensate for weak grip strength. Proper lighting and the use of personal glasses were recommended. Inpatients were trained to hold the dentures in a safe manner, clean the mucosal surface of the dentures, and direct brushing along the tooth axis. If considered helpful, the use of special tools, such as single tuft brushes, interdental brushes, triple-headed toothbrushes, or denture brushes was demonstrated, and training given. Practical training was continued until the inpatients mastered the techniques. This IndOHCT was conducted in a collaborative, patient-centered, and motivational manner to empower the inpatients to perform oral hygiene at their individual optimum levels.

Inpatients in the CG did not receive IndOHCT during the study period. To remedy this disadvantage, similar training was provided after the completion of data collection in this group.

The intervention lasted between 6.5 and 18 min. It lasted longer in subjects with residual dentition than in edentulous subjects and those without dentures.

During hospitalization, all the inpatients performed self-controlled oral hygiene. No oral hygiene-related instructions were given to the medical staff.

### 2.4. Collection of Geriatric Assessment Data

Geriatric assessment data were collected from medical records. As part of the daily routine, the MMSE by Folstein [43], Geriatric Depression Scale (GDS) by Yesavage [44,45], and Barthel Index (BI) [46] of all inpatients were assessed by the medical staff of the Department of Geriatric Medicine, with standardized and validated instruments.

The BI assesses basic everyday functions in inpatients with neurological or musculoskeletal impairment [46], evaluating the current degree of independence in self-sufficiency. A higher score indicates greater inpatient independence. In order to focus on competencies relevant to oral hygiene, Barthel’s criteria for personal hygiene (0–5 points), eating (0–5–10 points), and dressing (0–5–10 points) were included in further analyses. A maximum of 25 points was achievable, and fewer points indicated higher deficits.

The German version of the GDS was used to detect depressed mood and signs of depressive disorders. The scores range from 0 to 15. A score of 0–4 is within the normal range, 5–9 indicates mild depression, and ≥10 indicates moderate to severe depression [44]. We investigated whether depressive mood (GDS), cognitive impairment (MMSE), and limitations of independence (BI) influenced the ability to perform good oral hygiene.

### 2.5. Statistical Data Analysis

Data were collected using Excel 2016 (Microsoft Corporation, Redmond, WA, USA) and statistically analyzed using IBM SPSS Statistics for Windows, version 22.0 (IBM Corp., Armonk, NY, USA). Descriptive statistics were used to calculate numbers, mean values, and standard deviations. The *t*-test for the analysis of mean values and Fisher’s test for categorical data were applied. The effect size (ES; Cohen’s d) was calculated to demonstrate the effect of IndOHCT on plaque reduction. The ES was categorized as small (≤0.4), medium (0.5–0.7), or large (≥0.8) [47]. The level of significance was set at *p* ≤ 0.05, and no correction for multiple analyses was applied.

## 3. Results

### 3.1. Study Sample at Baseline

Inpatient characteristics of the IG and CG at baseline are presented in Table 1. The inpatients were aged between 63 and 93 years (mean age, 81.1 ± 7.1 years). The inpatients were grouped by age according to the age categories of the population-based representative fifth German oral health study [5]. The subgroup of inpatients aged < 75 years comprised only 17.8% of the study sample.

One fifth were male. In the IG, 13 inpatients had complete dentures in the mandible and maxilla. Another 6 inpatients were partially edentulous in the maxilla and had single dentures and 18 inpatients had removable partial dentures. In the mandible, another 3 inpatients had single dentures and 18 inpatients in the IG wore removable partial dentures.

There were also 13 edentulous inpatients in the CG. All wore dentures in the maxilla, but one of these patients lost his single denture of the mandible before hospitalization. Furthermore, another 6 inpatients had single dentures in the maxilla and 17 patients had removable partial dentures. In the mandible, another 19 patients of the CG had removable partial dentures. On average, all inpatients possessed 8.4 ± 8.4 and dentate inpatients 11.8 ± 7.6 remaining teeth.

There was no significant difference in oral health parameters between the groups at baseline, except the DHI and TmQHI.

The plaque values at baseline are shown in Table 2. At T0, the mean overall DHI in the IG (9.2 ± 5.7) was slightly lower than in the CG (11.1 ± 5.2); however, the difference was not statistically significant.

In younger inpatients, the DHI scores were significantly higher in the CG (*p* = 0.047) than in the IG. Inpatients with partial dentures in the maxilla (*p* = 0.007) had significantly higher DHI scores. At baseline, inpatients with mandibular partial dentures had significantly higher TmQHI scores in the IG (*p* = 0.031) than in the CG. No significant differences in DHI scores were detected between the complete denture wearers in either group.

### 3.2. Evaluation of oral Hygiene Status between the Beginning (T0) and End (T1a) of Hospitalization

Differences in plaque (TmQHI and DHI) reduction between T0 and T1a are presented in Table 3. No significant plaque reduction on the teeth or dentures was observed between T0 and T1a.

Age had a high ES on the DHI. Younger inpatients removed more plaque than older inpatients; however, the difference was not statistically significant (ES = 1.3; *p* = 0.133).

Sex, number of natural teeth, and PSI score had no significant influence on plaque reduction on teeth and dentures.

The BI total score had no significant effect on the DHI or TmQHI scores. Nevertheless, inpatients with high scores in the reduced BI scores removed significantly more plaque on dentures than those with lower scores (ES = 0.8; *p* = 0.029).

GDS and MMSE scores had no influence on the DHI score. None of the geriatric assessment tests were significantly associated with the TmQHI score.

### 3.3. Evaluation of Oral Hygiene Status before and after Autonomous Tooth Brushing and Denture Cleaning (T1a-T1b)

Table 4 demonstrates the teeth and denture plaque differences between T1a and T1b. Regardless of whether they received IndOHCT, both groups had reduced plaque on the teeth and dentures.

Inpatients in the IG showed more effective plaque reduction than the controls. Plaque reduction on dentures did not reach statistical significance in the direct comparison between the two groups (*p* = 0.093). Regardless of group membership, inpatients aged ≥ 75 years reduced significantly (*p* = 0.044) more denture plaque than younger inpatients, with a high ES (0.7).

In women, plaque reduction on teeth was more effective in the IG than in the CG (*p* < 0.001). Plaque reduction also improved in males; however, there was no significant difference between the groups.

Inpatients in the IG with 1–9 natural teeth removed more plaque than those in the CG (*p* < 0.001) and those with 10–28 natural teeth (ES = 0.8; *p* = 0.026). The number of natural teeth had no influence on the DHI score.

The PSI score demonstrated a significant effect on plaque reduction on the teeth (ES = 0.9; *p* = 0.02). In the IG, inpatients with gingivitis and periodontitis removed more plaque from the teeth than periodontally healthy inpatients. However, the PSI score had no effect on denture hygiene.

In the IG, plaque removal was significantly (*p* < 0.001) more effective in inpatients with higher GDS scores than in those with lower GDS scores, with a medium ES (0.6). In contrast, the CG showed the opposite effect. Inpatients with higher GDS scores reduced plaque on the teeth less effectively. However, these findings were not statistically significant. The GDS score had no influence on the DHI score.

The MMSE score significantly influenced denture hygiene in the entire study sample between T1a and T1b, with a medium ES (−0.5; *p* = 0.021). Inpatients with higher MMSE scores had significantly fewer plaques than those with lower scores (ES = −0.5; *p* = 0.021). The MMSE score had no influence on the TmQHI score. Between T1a and T1b, the BI score had no influence on the TmQHI and DHI scores.

## 4. Discussion

Our findings confirm that geriatric inpatients have a poor oral health status [9,48,49,50]. Oral cleanliness is required for maintaining or improving oral health. The oral health and oral hygiene status of hospitalized older inpatients are often poor [9,51]. Individual oral health care training could be effective, especially in short-term outcomes [52]. Nevertheless, studies on the effect of training to improve oral and denture hygiene in hospitalized older inpatients, even over a short period of time, are scarce [53]. Gibney et al. demonstrated an improvement in oral hygiene with a seven-day intervention under the guidance of an oral health therapist or trained nurse. There was a significantly higher proportion of inpatients (35–37%) whose oral cleanliness improved from unhealthy to healthy after the seven-day intervention than during the pre-intervention phase (17%). The TmQHI of the inpatients in the IG of the present study also improved by 35.5% between T1a and T1b, consistent with the findings of Gibney et al.

### 4.1. Evaluation of Oral Hygiene Status between the Beginning (T0) and end (T1a) of Hospitalization

Despite extensive IndOHCT in the IG at baseline, there were no significant differences between the IG and CG in plaque reduction on teeth and dentures. It can be speculated that inpatient self-motivation to maintain good oral hygiene was low. One possible reason for the stagnation of plaque scores between T0 and T1a could be that more focus was being placed on the acute inpatients’ general diseases, and not on oral hygiene. Consequently, dental and prosthetic care received little attention. Presumably, inpatients focus to increase their BI score by mobility training with the aim to become fit faster so that they can leave the hospital sooner. They invest their energy into mobility training by walking and climbing stairs [54]. Therefore, oral hygiene becomes less of a focus for the inpatients. Moreover, these efforts further reduce the motivation for oral hygiene.

Younger geriatric inpatients removed denture plaque more effectively than older inpatients but without statistical significance. At older ages, fine motor skills decrease, impairing practical competencies, such as oral hygiene, which was confirmed by Grönbeck Lindén et al. and Curreri et al. [55,56]. Elderly inpatients are at a risk of poor oral hygiene. Only approximately 33.4% of older people aged over 85 years are still fully capable of performing sufficient oral hygiene [5].

Sex, number of natural teeth, PSI score, and geriatric assessment tests had no significant influence on the TmQHI score between T0 and T1a. Nevertheless, the findings revealed a possible relationship between geriatric assessment and the DHI score. Activities of daily living measured by the total BI score had no significant effect on the DHI or TmQHI scores. However, the reduced sub-scores revealed a significant and high effect. High scores on the eating, personal hygiene, and dressing criteria were useful in predicting high denture cleaning efficacy. These findings are consistent with those of other studies where high BI scores were associated with good dental and denture hygiene [57].

### 4.2. Evaluation of Oral Hygiene Status before and after Autonomous Tooth Brushing and Denture Cleaning (T1a-T1b)

Between T1a and T1b, geriatric inpatients in both groups effectively removed plaque on the teeth and dentures. This suggests that the mere presence of a quietly observing caregiver may lead to improved plaque removal. Supervision clearly places the focus at that moment on the performance of oral hygiene. The more effective plaque removal on natural teeth in the IG could be explained by the received IndOHCT. Inpatients were able to perform more effective cleaning techniques that they were taught using their personal toothbrushing products and for which training was given. Geriatric inpatients who received IndOHCT achieved increased plaque reduction, achieving a significantly lower TmQHI score. Thus, oral and denture hygiene is improvable through supervision and implementation of IndOHCT. These findings are consistent with those of other studies [21,58]. Nakre and Harikiran summarized the effectiveness of a wide range of oral education programs on plaque reduction. The programs were more effective in short-term studies. Almas et al. investigated plaque reduction in chronically ill inpatients who received oral hygiene instructions and were re-evaluated after a seven-day period. There was more than 47% reduction in plaque scores. These findings are approximately consistent with those of the present study, which observed a 35.5% plaque reduction on natural teeth and 58.5% improvement in the DHI score in the IG.

Inpatients with 1–9 residual teeth benefited significantly more from IndOHCT, succeeding in removing more dental plaque than those with more residual teeth. Single or gap-bounded teeth are usually more difficult to clean than complete dentition, especially on approximal surfaces. A dentition with gaps requires the learning of new techniques whereas for an almost complete dentition, lifelong routine techniques can be maintained. Therefore, during IndOHCT, special attention was given to inpatients with reduced numbers of teeth to exercise individualized cleaning techniques.

Geriatric inpatients with healthy periodontal tissues or gingivitis also benefited more from IndOHCT than those with periodontitis. Presumably, inpatients with periodontitis would not have practiced effective oral hygiene for a long time. They were invited to learn fundamentally new techniques that could be perceived as very difficult. In addition, pain, loose teeth, and bleeding may have hampered the brushing process. In contrast, inpatients who already had good oral hygiene only had to change their habits slightly. Often, only small changes in brushing techniques were sufficient to improve plaque reduction.

The MMSE score demonstrated a medium effect on denture hygiene. Inpatients with lower MMSE scores removed more plaque on dentures than did those with higher MMSE scores. They almost halved the DHI scores of the baseline examinations. Due to the very limited inclusion criteria, which excluded inpatients with an MMSE score < 24 from the study, conclusions regarding the relationship between MMSE and plaque scores cannot be reliably drawn. Mental disorders are a risk indicator for poor oral health. Tooth decay, edentulism, and poor denture and oral hygiene are more prevalent in older adults with cognitive impairment than in those who are cognitively healthy [59].

Nevertheless, programs to improve oral health should also be implemented in hospitals. Many multimorbid individuals or older people requiring care take advantage of dental assistance irregularly and complaint-based [60]. As many studies, including the present study, show, older people in need of care sometimes have desolate dental status. Only 22.5% of the elderly (aged ≥ 75 years) in Germany who are in need of care perform oral hygiene without limitations, while 42.6% are no longer able to perform adequate oral hygiene [5]. Hospitalization may be the only chance for medical staff to routinely examine the oral cavity and detect oral health neglect early.

Comprehensive medical and dental preventive strategies for older people aiming to increase their quality of life should include individualized oral healthcare training. This study clearly revealed that the oral hygiene of geriatric inpatients can be improved by individualized supervised hands-on training on teeth and denture cleaning. Thus, both hypotheses could be partially confirmed: (1) First, the findings confirmed the effectiveness of IndOHCT in geriatric inpatients. IndOHCT improved the oral hygiene status of geriatric inpatients, and this is in line with the findings of other studies, showing that IndOHCT programs can be successful [61]. Nevertheless, the present study showed that the intervention alone did not improve oral hygiene. Only additional observation during tooth and denture care could lead to significant plaque reduction. Thus, supervision is another important element for improving oral hygiene in geriatric inpatients. (2) Second, age, sex, and the oral and geriatric health status of geriatric inpatients play a minor role in the older person’s capacity to remove plaque on teeth and dentures.

In comparison with the present study, an intervention study by Frenkel et al. also clearly demonstrated that oral health education of non-dental staff can improve the oral health of institutionalized elders [62]. Dental care awareness needs to be increased in geriatric wards. Literature reveals that preventing and early detection of oral disease can improve the general well-being and conserve financial resources [63]. Education of nursing staff is crucial for improving oral health in geriatric inpatients [22,64,65]. However, it is not only the nursing staff that should be better trained in oral hygiene. Supervision in performing oral hygiene can also be performed by untrained staff and relatives, which could be a cost-effective possibility. Therefore, they should be integrated into intervention programs to ensure improved oral hygiene, even after hospitalization [66]. In addition, there is often a lack of oral hygiene products in geriatric wards or they are from low quality, which can hinder the execution of IndOHCT by nursing stuff. Further research is necessary to find strategies that will address the problem on a population level and that would be cost-effective, realistic, and sustainable.

### 4.3. Strengths and Limitations of the Study

The present study contributes to the evidence that IndOHCT is effective for enhancing tooth and denture brushing skills in geriatric inpatients during hospitalization. Furthermore, the narrow selection of geriatric inpatients with precise group assignment according to dentition status, wearing of prostheses, and sex reduced selection bias and fostered good comparability between groups. Bias due to various general diseases and associated general conditions could not be avoided. Furthermore, caries experience in the study sample was consistent with the national average (older patients in need of care, 25.4 vs. 24.6 DMFT) [5]. Nevertheless, comparability with the fifth German oral health study must be considered with caution, since the present study also included younger elders and those without care needs. Only 28.9% of the inpatients required daily care; however, all had multimorbidity and acute medical conditions. In contrast to the fifth German oral health study, the geriatric inpatients in this study had a lower prevalence of edentulism (28.9% vs. 53.7%) and had more natural teeth on average (8.4 vs. 5.9). Moreover, periodontal disease was less common in the present study (57.8% vs. 90%).

It is well known that learning new skills can be inhibited by existing habits [67]. Strategies to enhance behavior changes in oral health should therefore be based on behavioral theory [68]. In this study, the InOHCT empowered the patients to enhance their tooth-brushing skills by intensive hands-on training in individual denture and toothbrushing techniques and theoretical face-to-face instruction on the importance of good oral hygiene.

This study had some limitations. First, it was conducted in a hospital setting. An examination at the inpatient’s bed does not provide comfort or the ability to position the inpatient to sufficiently inspect the dorsal areas of the oral cavity. Plaque might have been overlooked in areas that were difficult to inspect. In addition, the study was conducted exclusively at a single site; therefore, generalization and transfer to other sites is limited.

Second, only inpatients with an MMSE score of at least 24 points were included in the study. Therefore, the weak relationship between cognitive impairment and oral health might have selection bias. To investigate the ability to learn effective oral hygiene, inpatients with moderate and severe cognitive impairment should be included in further investigations.

Third, the influence of medication was not considered, which could lead to dry mouth and poorer plaque values [69].

Fourth, the study covered an average hospitalization period of 12.4 days; therefore, it is unclear whether the effect of IndOHCT is sustained after hospitalization. The literature reveals that short-time observations failed to have essential long-term effects [69]. Long-term studies would provide more reliable data regarding the effectiveness and sustainability of IndOHCT.

Therefore, due to the limited number of studies, further research on the effectiveness of tooth and denture cleaning training in geriatric wards is of particular interest, as well as long-term follow-up studies evaluating the sustained success of IndOHCT in geriatric inpatients.

## 5. Conclusions

Within the limitations of the present study, it was revealed that sex, age, and oral and geriatric health status had only a marginal influence on plaque removal on teeth and dentures of geriatric inpatients. However, it was demonstrated that IndOHCT has a positive effect on the competence of geriatric inpatients to remove plaque from teeth and dentures over a short-term observation period. Therefore, it is recommended to provide IndOHCT to geriatric inpatients during hospitalization.

## Figures and Tables

**Table 1:** Study population’s age, sex, oral status, and geriatric assessment at baseline (T0).

		Total			Control Group			Intervention Group		
		All	Age < 75 Years	Age ≥ 75 Years	All	Age < 75 Years	Age ≥ 75 Years	All	Age < 75 Years	Age ≥ 75 Years
Patients, *n* (%)		90 (100)	16 (17.8)	74 (82.2)	45 (50)	9 (10)	36 (40)	45 (50)	7 (7.8)	38 (42.2)
Sex, *n* (%)	Male ♂	18 (20)	6 (6.7)	12 (13.3)	9 (20)	2 (2.2)	7 (7.8)	9 (10)	4 (4.4)	5 (5.6)
	Female ♀	72 (80)	10 (11.1)	62 (68.9)	36 (40)	7 (7.8)	29 (32.2)	36 (40)	3 (3.3)	33 (36.7)
Age, mean ± SD		81.1 ± 7.1	69.2 ± 3.8	83.6 ± 4.7	79.9 ± 6.8	69.3 ± 3.5	82.5 ± 4.5	82.2 ± 7.3	69 ± 4.4	84.6 ± 4.4
Edentulism, n (%)		26 (28.9)	4 (4.4)	22 (24.4)	13 (14.4)	3 (3.3)	10 (11.1)	13 (14.4)	1 (1.1)	12 (13.3)
Number of teeth, mean ± SD		8.4 ± 8.4	9.6 ± 8.0	8.1 ± 8.5	9.5 ± 8.6	9.0 ± 8.3	9.6 ± 8.8	7.2 ± 8.0	10.4 ± 8.2	6.6 ± 8.0
DMFT, mean ± SD		25.4 ± 3.7	24.5 ± 3.4	25.5 ± 3.8	25.1 ± 3.6	25.0 ± 3.1	25.1 ± 3.7	25.6 ± 3.9	23.9 ± 4.0	25.9 ± 3.8
DT, mean ± SD		2.0 ± 2.8	3.2 ± 4.0	1.7 ± 2.5	2.1 ± 2.9	3.8 ± 4.8	1.7 ± 2.2	1.9 ± 2.8	2.5 ± 3.3	1.7 ± 2.7
PSI max, mean ± SD		2.5 ± 1.1	2.75 ± 1.0	2.5 ± 1.1	2.5 ± 0.9	2.7 ± 0.8	2.5 ± 1.0	2.5 ± 1.2	2.8 ± 1.2	2.5 ± 1.3
Dentures, n (%)		74 (82.2)	13 (13.3)	61 (67.8)	37 (41.1)	8 (8.9)	29 (32.2)	37 (41.1)	5 (5.6)	32 (35.6)
TmQHI, mean ± SD		3.0 ± 0.9	3.0 ± 0.8	3.0 ± 1.0	2.9 ± 1.0	2.94 ± 1.1	2.9 ± 1.0	3.1 ± 0.8	3.1 ± 0.3	3.1 ± 0.9
DHI, mean ± SD		10.1 ± 5.5	9.8 ± 6.7	10.2 ± 5.3	11.1 ± 5.2	12.6 ± 6.6	10.6 ± 4.9	9.2 ± 5.7	5.2 ± 4.3	9.8 ± 5.7
Geriatric assessment, mean ± SD	GDS	3.6 ± 2.5	5.1 ± 3.7	3.3 ± 2.1	3.6 ± 2.5	5.9 ± 3.6	3.1 ± 1.8	3.6 ± 2.6	4.1 ± 3.8	3.5 ± 2.4
	MMS	26.5 ± 1.8	26.9 ± 1.9	26.5 ± 1.8	26.9 ± 1.7	27.4 ± 1.8	26.7 ± 1.6	26.2 ± +1.9	26.1 ± 1.9	26.2 ± 1.9
	Barthel	62.3 ± 17.7	61.6 ± 17.5	62.5 ± 17.8	62.7 ± 16.9	59.4 ± 19.1	63.5 ± 16.5	62.0 ± 18.6	64.3 ± 16.2	61.6 ± 19.1

**Table 2:** Turesky-modified Quigley–Hein index (TmQHI) and denture hygiene index (DHI) at baseline (T0).

Variables			TmQHI At Baseline					DHI at Baseline				
			N Patients CG/IG	All	CG	IG	*p*-Value	N Patients CG/IG	All	CG	IG	*p*-Value
				Mean ± SD	Mean ± SD	Mean ± SD			Mean ± SD	Mean ± SD	Mean ± SD	
	All		32/32	3.0 ± 0.9	2.9 ± 1.0	3.1 ± 0.8	0.275	37/37	10.1 ± 5.5	11.1 ± 5.2	9.2 ± 5.7	0.146
Age		<75	6/6	3.0 ± 0.8	2.9 ± 1.1	3.1 ± 0.3	0.693	8/5	9.8 ± 6.7	12.6 ± 6.6	5.2 ± 4.3	**0.047**
		≥75	29/32	3.0 ± 1.0	3.0 ± 1.0	3.1 ± 0.9	0.318	26/26	10.2 ± 5.3	10.6 ± 4.9	9.8 ± 5.7	0.554
Sex		Male ♂	6/7	3.2 ± 0.8	3.2 ± 0.9	3.2 ± 0.8	0.999	8/6	10.5 ± 3.4	11.1 ± 3.2	9.7 ± 3.8	0.451
		Female ♀	26/25	2.9 ± 0.9	2.8 ± 1.0	3.1 ± 0.8	0.255	29/31	10.0 ± 5.9	11.0 ± 5.7	9.1 ± 6.0	0.207
Number of teeth		0	0	-	-	-	-	13/13	11.9 ± 5.7	12.1 ± 4.8	11.7 ± 6.7	0.868
		1–9	11/18	3.2 ± 0.9	3.3 ± 0.9	3.2 ± 0.9	0.768	11/16	10.0 ± 4.9	11.4 ± 5.8	9.1 ± 4.0	0.246
		10–19	12/8	3.0 ± 0.7	2.9 ± 0.9	3.1 ± 0.4	0.662	13/8	8.0 ± 5.5	9.8 ± 5.4	5.3 ± 4.9	0.068
		≥20	9/6	2.6 ± 1.1	2.3 ± 1.1	3.1 ± 1.0	0.210	0/0	-	-	-	-
Type of prostheses	upper jaw	partial	17/18	3.0 ± 1.2	3.1 ± 1.1	2.8 ± 1.3	0.495	17/18	4.9 ± 2.9	6.3 ± 3.0	3.7 ± 2.2	**0.007**
		total	-	-	-	-	-	19/19	6.2 ± 2.1	6.8 ± 2.3	5.6 ± 3.4	0.224
	lower jaw	partial	19/18	3.1 ± 1.0	2.8 ± 1.0	3.5 ± 0.9	**0.031**	19/18	5.0 ± 3.6	5.8 ± 4.1	4.3 ± 2.7	0.275
		total	-	-	-	-	-	12/16	6.5 ± 3.1	6.4 ± 3.0	6.5 ± 3.2	0.893
PSI		0	1/4	2.8 ± 0.4	2.7 ± 0.0	2.8 ± 0.5	0.812	1/3	10.8 ± 4.0	15.0 ± 0.0	9.3 ± 3.5	0.297
		1–2	13/9	2.9 ± 1.0	2.85 ± 1.0	3.0 ± 1.1	0.711	10/7	7.8 ± 5.1	9.4 ± 5.4	5.6 ± 4.0	0.133
		3–4	18/19	3.1 ± 0.9	2.9 ± 1.0	3.2 ± 0.7	0.252	13/14	9.8 ± 5.4	11.0 ± 5.8	8.6 ± 4.9	0.264

*t*-test between CG and IG; significant values are displayed in bold.

**Table 3:** Plaque reduction and effect of intervention measured by the TmQHI and DHI between T0 and T1a.

		Plaque Reduction between T0 and T1a (Mean Duration, 12.4 Days)									
Variables		TmQHI-Difference					DHI-Difference				
		N CG/IG	All Mean ± SD	CG Mean ± SD	IG Mean ± SD	CG vs. IG Effect Size (*p*-Value)	N CG/IG	All Mean ± SD	CG Mean ± SD	IG Mean ± SD	CG vs. IG Effect Size (*p*-Value)
	All	32/32	0.1 ± 0.6	0.0 ± 0.5	0.1 ± 0.6	0.2 (0.333)	37/37	−0.3 ± 4.3	0.0 ± 4.1	−0.7 ± 4.6	−0.2 (0.473)
Age	<75	6/6	−0.1 ± 0.8	−0.2 ± 0.7	0.0 ± 0.8	0.3 (0.642)	8/5	1.3 ± 5.1	2.4 ± 5.4	−0.4 ± 4.6	**−0.6** (0.363)
	≥75	26/26	0.1 ± 0.5	0.0 ± 0.5	0.2 ± 0.6	0.2 (0.409)	29/32	−0.7 ± 4.1	−0.6 ± 3.4	−0.8 ± 4.7	0 (0.904)
	ES (*p*-value) (<75 vs. ≥75)	-	0.3 (0.386)	0.3 (0.409)	0.3 (0.680)	-	-	1.3 * (0.133)	**−0.7** (0.064)	−0.1 (0.878)	-
Sex	Male ♂	6/7	0.0 ± 0.4	0.2 ± 0.4	−0.1 ± 0.4	−0.7 (0.219)	8/6	−0.4 ± 2.5	0.0 ± 1.2	−1.0 ± 3.7	−0.4 (0.487)
	Female ♀	26/25	0.1 ± 0.6	−0.1 ± 0.6	0.2 ± 0.6	0.4 (0.139)	29/31	−0.3 ± 4.7	0.0 ± 4.6	−0.7 ± 4.8	−0.1 (0.578)
	ES (*p*-value) (♂ vs. ♀)	-	0.2 (0.691)	**−0.6** (0.396)	**0.6** (0.198)	-	-	<0.1 (0.931)	<0.01 (0.983)	0.3 (0.866)	-
Number of teeth	0	0/0	-	-	-	-	13/13	−0.6 ± 3.3	−0.3 ± 3.5	−0.9 ± 3.3	−0.2 (0.645)
	1–9	11/18	0.2 ± 0.6	0.1 ± 0.6	0.3 ± 0.6	0.3 (0.533)	11/16	0.4 ± 4.7	0.1 ± 4.5	0.6 ± 4.9	0.1 (0.777)
	10–19	13/9	−0.1 ± 0.6	0.0 ± 0.5	−0.2 ± 0.7	−0.3 (0.511)	13/8	−1.0 ± 5.0	0.3 ± 4.6	−3.0 ± 5.4	**−0.7** (0.149)
	≥20	8/5	0.0 ± 0.5	−0.2 ± 0.5	0.2 ± 0.2	1.1 * (0.159)	0/0	-	-	-	-
	ES (*p*-value) (0–9 vs. 10–28)	-	**0.5** (0.069)	0.3 (0.366)	**0.5** (0.175)	-	-	0.2 (0.447)	−0.1 (0.762)	**0.6** (0.114)	-
PSI	0	1/4	−0.3 ± 0.6	0.0 ± 0.0	−0.3 ± 0.7	(0.688)	1/3	0.5 ± 1.9	0.0 ± 0.0	0.7 ± 2.3	(0.826)
	1–2	13/9	0.1 ± 0.6	0.1 ± 0.6	0.2 ± 0.6	0.2 (0.771)	10/7	−0.3 ± 4.7	−0.6 ± 2.0	0.1 ± 7.2	0.1 (0.759)
	3–4	18/19	0.1 ± 0.6	−0.1 ± 0.5	0.2 ± 0.6	0.5 (0.112)	13/14	−0.2 ± 5.3	0.9 ± 5.8	−1.2 ± 4.8	−0.4 (0.323)
	ES (*p*-value) (0–2 vs. 3–4)	-	<0.1 (0.961)	0.4 (0.311)	−0.3 (0.422)	**-**	-	0.1 (0.956)	−0.3 (0.454)	0.3 (0.501)	-
MMS	<MAV	10/19	0.2 ± 0.6	0.2 ± 0.7	0.1 ± 0.6	−0.2 (0.802)	14/24	−0.6 ± 4.7	0.1 ± 3.2	−1.0 ± 5.5	−0.3 (0.493)
	≥MAV	22/13	0.0 ± 0.6	−0.1 ± 0.4	0.1 ± 0.6	0.4 (0.218)	23/13	0.0 ± 3.9	0.0 ± 4.6	−0.1 ± 2.5	0 (0.956)
	ES (*p*-value) (<MAV vs. ≥MAV)	-	−0.3 (0.227)	**−0.5** (0.146)	0 (0.964)	-		0.1 (0.553)	0 (0.959)	0.2 (0.553)	-
GDS	<MAV	18/17	0.1 ± 0.6	0.0 ± 0.5	0.2 ± 0.7	0.3 (0.450)	22/20	−0.3 ± 3.9	−0.3 ± 4.0	−0.4 ± 4.0	0 (0.950)
	≥MAV	14/15	0.0 ± 0.6	−0.1 ± 0.6	0.1 ± 0.5	0.4 (0.534)	15/17	−0.4 ± 4.9	0.5 ± 4.2	−1.1 ± 5.4	−0.3 (0.367)
	ES (*p*-value) (<MAV vs. ≥MAV)	-	−0.2 (0.443)	−0.2 (0.599)	−0.2 (0.557)	-	-	0.0 (0.949)	0.2 (0.594)	−0.1 (0.622)	-
Reduced BI	≤20	14/12	0.1 ± 0.5	0.0 ± 0.6	0.3 ± 0.4	0.6 (0.149)	16/12	1.1 ± 4.5	0.6 ± 4.1	1.7 ± 5.1	0.2 (0.557)
	>20	18/20	0.0 ± 0.6	0.0 ± 0.6	0.0 ± 0.7	0 (0.809)	21/25	−1.2 ± 4.0	−0.4 ± 4.0	−1.8 ± 4.0	−0.4 (0.241)
	ES (*p*-value) (≤20 vs. >20)	-	0.2 (0.382)	0 (0.936)	**0.5** (0.245)	-	**-**	**0.5 (0.028)**	0.2 (0.442)	**0.8 (0.029)**	-

*t*-test between CG and IG; ES, effect size (Cohen’s d); * high and middle effect sizes; significant values are displayed in bold.

**Table 4:** Plaque reduction and effect of intervention measured by the TmQHI and the DHI between T1a and T1b.

		Effect of Intervention T1a-T1b									
			TmQHI-Difference					DHI-Difference			
		N CG/IG	All Mean ± SD	CG Mean ± SD	IG Mean ± SD	CG vs. IG Effect Size (*p*-Value)	N CG/IG	All Mean ± SD	CG Mean ± SD	IG Mean ± SD	CG vs. IG Effect Size (*p*-Value)
	All	32/32	0.9 ± 0.5	0.7 ± 0.3	1.1 ± 0.5	**1.0 (<0.001)**	37/37	4.6 ± 4.0	3.8 ± 3.5	5.4 ± 4.4	0.4 (0.093)
Age	<75	6/6	0.9 ± 0.4	0.7 ± 0.2	1.2 ± 0.5	1.3 * (0.046)	8/5	2.5 ± 2.6	2.1 ± 1.7	3.2 ± 3.8	0.4 (0.498)
	≥75	26/26	0.9 ± 0.5	0.7 ± 0.3	1.1 ± 0.5	1.0 * (0.001)	29/32	5.0 ± 4.1	4.2 ± 3.8	5.7 ± 4.4	0.4 (0.175)
	ES (*p*-value) (<75 vs. ≥75)	-	0.0 (0.952)	0.0 (0.908)	−0.2 (0.878)	**-**	-	**0.7 (0.044)**	**0.7** (0.135)	**0.6** (0.241)	-
Sex	Male ♂	6/7	1.0 ± 0.5	0.8 ± 0.3	1.1 ± 0.7	**0.6** (0.218)	8/6	4.4 ± 3.6	4.0 ± 4.3	5.0 ± 2.6	0.3 (0.626)
	Female ♀	26/25	0.9 ± 0.5	0.7 ± 0.3	1.1 ± 0.5	1.0 * (<0.001)	29/31	4.6 ± 4.1	3.7 ± 3.4	5.4 ± 4.7	0.4 (0.114)
	ES (*p*-value) (♂ vs. ♀)	-	−0.2 (0.703)	−0.3 (0.619)	0.0 (0.997)	**-**	-	0.1 (0.887)	−0.1 (0.848)	0.1 (0.833)	-
Number of teeth	0	0/0	-	-	-	**-**	13/13	5.2 ± 4.0	3.7 ± 2.6	6.8 ± 4.8	0.8 * (0.051)
	1–9	11/18	1.1 ± 0.5	0.7 ± 0.3	1.3 ± 0.5	1.5 * (<0.001)	11/16	4.6 ± 4.0	4.3 ± 3.7	4.9 ± 4.3	0.2 (0.707)
	10–19	13/9	1.0 ± 0.4	0.9 ± 0.3	1.1 ± 0.4	0.6 (0.094)	13/8	3.7 ± 4.0	3.5 ± 4.4	4.0 ± 3.6	0.1 (0.775)
	≥ 20	8/5	0.5 ± 0.2	0.5 ± 0.2	0.6 ± 0.2	0.5 (0.617)	0/0	-	-	-	-
	ES (*p*-value) (0–9 vs. 10–28)	-	**0.6 (0.021)**	−0.1 (0.72)	0.8 * (0.026)	-	-	0.3 (0.227)	0.1 (0.689)	0.4 (0.329)	-
PSI	0	1/4	1.2 ± 0.8	0.7 ± 0.0	1.3 ± 0.8	1.1 * (0.532)	1/3	4.3 ± 4.8	5.7 ± 4.7	0.0 ± 0.0	(0.408)
	1–2	13/9	1.0 ± 0.6	0.7 ± 0.4	1.4 ± 0.5	1.5 * (0.001)	10/7	3.5 ± 3.3	3.9 ± 3.7	2.9 ± 1.1	−0.4 (0.543)
	3–4	18/19	0.8 ± 0.4	0.7 ± 0.3	1.0 ± 0.4	0.8 * (0.016)	13/14	4.7 ± 4.3	4.1 ± 4.4	5.2 ± 4.3	0.3 (0.505)
	ES (*p*-value) (0–2 vs. 3–4)	-	0.4 (0.12)	<0.01 (0.978)	0.9 * (0.02)	**-**	-	0.3 (0.372)	−0.1 (0.754)	−0.4 (0.375)	-
MMS	<MAV	10/19	1.0 ± 0.5	0.8 ± 0.3	1.2 ± 0.5	1.0 * (0.037)	14/24	5.6 ± 3.7	4.4 ± 2.8	6.3 ± 4.1	0.5 (0.139)
	≥MAV	22/13	0.8 ± 0.4	0.7 ± 0.3	1.1 ± 0.5	1.0 * (0.003)	23/13	3.5 ± 4.1	3.4 ± 3.9	3.6 ± 4.5	0.0 (0.877)
	ES (*p*-value) (<MAV vs. ≥MAV)	-	−0.4 (0.096)	−0.3 (0.457)	−0.2 (0.725)	**-**	-	**−0.5 (0.021)**	−0.3 (0.393)	**−0.6** (0.074)	-
GDS	<MAV	18/17	0.9 ± 0.4	0.8 ± 0.3	1.0 ± 0.5	0.5 (0.145)	22/20	4.4 ± 3.9	4.1 ± 3.9	4.7 ± 4.0	0.2 (0.620)
	≥MAV	14/15	1.0 ± 0.5	0.6 ± 0.3	1.3 ± 0.5	1.7 * (<0.001)	15/17	4.8 ± 4.2	3.3 ± 3.1	6.1 ± 4.7	0.7 (0.061)
	ES (*p*-value) (<MAV vs. ≥MAV)	-	0.2 (0.607)	**−0.7** (0.07)	**0.6** (0.091)	**-**	-	0.1 (0.65)	−0.2 (0.529)	0.3 (0.331)	-
Reduced BI	≤20	14/12	0.9 ± 0.5	0.6 ± 0.3	1.1 ± 0.5	**1.2 (0.010)**	16/12	4.1 ± 2.9	3.9 ± 3.0	4.3 ± 2.9	0.1 (0.785)
	>20	18/20	1.0 ± 0.5	0.7 ± 0.3	1.2 ± 0.5	**1.2 (0.004)**	21/25	4.9 ± 4.6	3.7 ± 4.0	5.9 ± 4.9	**0.5** (0.102)
	ES (*p*-value) (≤20 vs. >20)	-	−0.2 (0.394)	−0.3 (0.377)	−0.2 (0.784)	**-**	-	−0.2 (0.411)	0.1 (0.821)	−0.4 (0.293)	-

*t*-test between CG and IG; ES, effect size (Cohen’s d); * high and middle effect sizes; significant values are displayed in bold.

## Data Availability

The datasets used and analyzed during the current study are available from the corresponding author on request. Informed consent was obtained from all subjects involved in the study.
